# Knockdown of Smurf1 Promotes Autophagy‐Dependent Ferroptosis in Acute Liver Failure by Inhibiting Ubiquitin‐Mediated Ulk1 Degradation

**DOI:** 10.1111/jcmm.71405

**Published:** 2026-10-29

**Authors:** Pei Shi, Mengyu Tao, Yuyu Zeng, Yuchen Peng, Xiaoping Wu

**Affiliations:** ^1^ Department of Infectious Diseases, the First Affiliated Hospital, Jiangxi Medical College Nanchang University Nanchang China

**Keywords:** acute liver failure, autophagy, ferroptosis, Smurf1, Ulk1

## Abstract

Treating acute liver failure (ALF) relies on rescuing dying hepatocytes and restoring liver regeneration. Ferroptosis contributes to ALF development, so regulating iron homeostasis or lipid peroxide production may be an effective treatment strategy. Ubiquitination can modify ferroptosis and is involved in liver diseases. The study aims to explore the mechanism of E3 ubiquitin ligase Smurf1 regulating ferroptosis in acute liver failure. Screened using proteomics, bioinformatics and experimental verification, we identified Smurf1, a ubiquitin‐related protein associated with ferroptosis. Functional experiments were used to verify that Smurf1 regulates hepatocyte ferroptosis and acute liver failure. Furthermore, ubiquitination assays, western blotting and Co‐IP experiments were applied to identify Ulk1 as a substrate of Smurf1. Further, we constructed the cell and animal models with silencing or overexpression of Smurf1 and Ulk1 to clarify the potential mechanism of Smurf1 on ferroptosis in ALF. Knockdown or overexpression of Smurf1 promoted or inhibited hepatocyte ferroptosis, respectively. In the animal model, knockdown of Smurf1 significantly aggravated the progression of ALF, suggesting that Smurf1 knockdown exacerbated liver failure by promoting hepatocyte ferroptosis. In conclusion, Smurf1 interacted with the new substrate Ulk1 and promoted its ubiquitin‐mediated degradation at the post‐translational level, and Smurf1 knockdown promotes ferroptosis in hepatocytes in a manner dependent on Ulk1‐mediated autophagy activation.

## 1 Introduction

Acute liver failure (ALF) is a severe injury syndrome caused by multiple factors, resulting in massive necrosis of hepatocytes and significant impairment of liver function [1]. Currently, there are no effective medical treatment methods available, and existing treatment strategies mainly focus on reducing hepatocyte death [2]. Cell death in the liver is primarily carried out through apoptosis or necrosis. Recently, several different forms of programmed cell death have been defined; among them, ferroptosis is a non‐apoptotic form of regulated cell death [3]. Excessive iron accumulation is a major feature of most liver diseases, and ferroptosis has attracted increasing attention in the field of liver disease due to the susceptibility of the liver to oxidative damage.

Ferroptosis is caused by the excessive accumulation of phospholipid peroxides, which are generated from the oxidation of polyunsaturated fatty acyl (PUFA) moieties in membrane phospholipids and is catalysed by iron‐dependent mechanisms [4]. After the occurrence of liver failure, a large number of cells die and cause abnormal accumulation of free iron. Excessive free iron induces oxidative stress and ferroptosis through the Fenton reaction, further aggravating hepatocyte death [5, 6]. Therefore, inhibiting ferroptosis can improve hepatocyte survival and suppress the progression of liver failure [7].

Ubiquitin‐proteasome system (UPS) is a widely existing protein modification and degradation pathway in cells [8, 9]. Among them, E3 ubiquitin ligases, which determine the specific recognition of target proteins, serve as key enzymes in the ubiquitination process [10, 11]. In liver diseases, UPS has been found to be involved in regulating the ferroptosis pathway. The protein stability of key regulatory molecules in the ferroptosis pathway, such as SLC7A11, GPX4, ACSL4, NCOA4 and DMT1, is strictly regulated by E3 ubiquitin ligases and deubiquitinating enzymes [12]. Ubiquitination is involved in the regulation of the hepatocyte ferroptosis pathway in liver diseases [13, 14, 15]. Smurf1 (Smad ubiquitination regulatory factor 1) is an E3 ubiquitin ligase that regulates the progression of various liver diseases by recognizing substrate proteins and mediating their ubiquitination modification [16, 17, 18]. Studies indicate that Smurf1 promotes the inhibition of hepatocellular carcinoma growth by regulating the ubiquitination of UVRAG [19]; Smurf1 can promote the ubiquitination and degradation of PPARγ, thereby alleviating hepatic steatosis [20, 21]. Although Smurf1 has been recognized to be associated with liver diseases, its specific molecular mechanism has not been fully elucidated, especially in acute liver failure.

Unc‐51 like autophagy activating kinase 1 (Ulk1) is an initiator of autophagy. The activation of the Ulk complex can further promote the formation of autophagosomes, while the inactivation of Ulk1 also leads to the termination of autophagy [22, 23]. Autophagy is a process involving the degradation of cellular components, such as dysfunctional proteins and damaged organelles, via lysosomes [24, 25]. It is an indispensable factor for maintaining cellular homeostasis. Previous studies have shown that the activation of autophagy degrades ferritin to release free iron, which in turn mediates the Fenton reaction, resulting in the accumulation of lipid ROS and the aggravation of ferroptosis [26, 27, 28, 29]. Therefore, autophagy is considered an upstream mechanism of ferroptosis, which induces ferroptosis by regulating cellular iron homeostasis and the production of reactive oxygen species (ROS).

In this study, we found that E3 ubiquitin ligase Smurf1, a protein associated with ferroptosis, is involved in regulating the ferroptosis process of hepatocytes, thereby influencing the progression of liver failure. To further investigate whether Smurf1 regulates hepatocyte ferroptosis through the autophagic pathway, we observed that Smurf1 knockdown maintains protein homeostasis by reducing the ubiquitination level of its substrate target protein Ulk1. This activation of the Ulk1‐mediated autophagic pathway then promotes the occurrence of hepatocyte ferroptosis and exacerbates the progression of liver failure. The Smurf1‐Ulk1 axis may serve as a potential therapeutic target for regulating ferroptosis in liver failure.

## 2 Methods

### 2.1 Animal Model

Male C57BL/6 mice (20‐25 g) obtained from Tianqin Biotechnology (Changsha, China) were intraperitoneally injected with lipopolysaccharide (LPS) (10 μg/kg) and D‐galactosamine (D‐GalN) (800 mg/kg) to establish an ALF model and were sacrificed at the specified time points. Blood and liver samples were taken for further research. The animal experiment was approved and supervised by the Laboratory Animal Committee of Nanchang University.

### 2.2 Biochemical Assay for AST/ALT and Haematoxylin and Eosin (H&E) Staining

Blood samples were collected from the retroorbital venous plexus and centrifugation at 3500 rpm for 10 min. Serum alanine transaminase (ALT) and aspartate aminotransferase (AST) were detected by Hitachi 7600 automatic biochemical analyser.

Liver tissues were placed in the fixing solution containing 4% paraformaldehyde (PFA). After 24 h, liver tissue was embedded in paraffin, cut into 4‐μm‐thick sections, deparaffinized, rehydrated and then stained with haematoxylin and eosin and stained with haematoxylin and eosin. The tissue sections were scanned with pathological slice scanner (Olympus, Tokyo, Japan). Finally, images were collected and analysed under an optical microscope (Zeiss, Germany).

### 2.3 Cell Culture and Live/Dead Cell Detection

The HepaRG cell was purchased from Pricella (Wuhan, China) and cultured with high glucose DMEM medium with 10% FBS (Gibco). We stained HepaRG cells with live/dead staining reagent (Solarbio, Beijing) for 30 min. Cell death was observed under an inverted fluorescence microscope (ZEISS, Germany).

### 2.4 Malondialdehyde, Glutathione and Iron Assay

The levels of malondialdehyde (MDA), glutathione (GSH) and Fe^2+^ in cell or tissue lysates were measured using detection kits from Beyotime (China), following the manufacturer's instructions.

### 2.5 Co‐Immunoprecipitation (Co‐IP) and Immunoprecipitation Coupled With Mass Spectrometry (IP/MS) Assay

The immunoprecipitation lysis buffer (Thermo Scientific, USA) was used to isolate proteins from HEK 293 T cells and HepaRG cells. Subsequently, the mixture was shaken on a shaker at 4°C for 30 min, then transferred to a centrifuge and centrifuged at 12,000 ***g*** for 30 min. The supernatant was collected and then incubated with 1 μg of the corresponding antibody overnight. The next day, the mixture was incubated with Protein A/G Magnetic Beads (MCE, USA) for 2 h, followed by washing 5–6 times with immunoprecipitation buffer. Finally, SDS‐PAGE protein loading buffer (2X) (Beyotime, China) was added for immunoblotting.

As described above, we first extracted total proteins from the cells, then incubated them with Smurf1 antibody, IgG antibody and Protein A/G Magnetic Beads for immunoprecipitation (IP) experiment. Finally, mass spectrometry (MS) was conducted to analyze the proteins that bind to Smurf1.

### 2.6 Western Blotting Analysis

Proteins were extracted by RIPA buffer (Beyotime, China). Samples were separated by SDS‐PAGE and then transferred to PVDF membranes. The membranes were blocked with skimmed milk powder, followed by incubation with primary antibodies and secondary antibodies. The protein bands were detected with Bio‐Rad chemiluminescence gel imaging equipment (Bio‐Rad, USA) and analysed with Image J 6.0 software.

### 2.7 Lentiviral Transfection

HepaRG cells were infected with lentiviral shRNA controls (sh‐NC), lentiviral shRNA‐Smurf1 (sh‐Smurf1), lentiviral shRNA‐Ulk1 (sh‐Ulk1), lentiviral overexpression controls (Oe‐Con) or lentiviral overexpression Smurf1 (Oe‐Smurf1) (HanBio, China) to knock down or overexpress the corresponding genes. The target sequences of the shRNAs are shown in Table 1.

**TABLE 1:** Target sequences of sh‐RNA.

Gene	Sequence
sh NC	TTCTCCGAACGTGTCACGT
sh Smruf1#1	GAGATGTTCCGTGTGATTA
sh Smurf1#2	CAGACCTACGAGAACATCG
sh Ulk1#1	GCTACAAGAAGATGGAGAA
sh Ulk1#2	CCAGAGATGTTCGTGATTA

### 2.8 Adeno‐Associated Virus (AAV) Infection

After successfully establishing a mouse model of liver failure, to downregulate the expression of Smurf1 in the liver region, we slowly injected AAV‐sh Smurf1 virus (titre: 6 × 10^10^ viral genomes, injection volume: 250 μL) via the tail vein and conducted a synchronous experiment using AAV‐sh Con as the control vector.

### 2.9 Statistical Analysis

For comparisons between two groups, an unpaired two‐tailed Student's *t*‐test was used; for comparisons involving more than two groups, one‐way or two‐way analysis of variance (ANOVA) was applied, followed by Tukey's post hoc test. Data analyses were performed using GraphPad Prism 8.0. All experimental data were independently repeated at least three times. A value of *p* < 0.05 was considered statistically significant.

## 3 Results

### 3.1 Smurf1 May Be a Protein Associated With Ferroptosis in Liver Injury

The results of ALT, AST and H&E staining indicated that the mouse model of ALF was successfully established (Figure 1A–C). Ferroptosis has been proven to be an important pathological mechanism in ALF. Glutathione depletion, Fe^2+^ accumulation and lipid peroxidation are key inducing factors of ferroptosis. As expected, there was a significant ferroptosis phenomenon in ALF mice (increased Fe^2+^ concentration and MDA content; decreased expression of Fth1 and Gpx4; Figure 1D–F).

**FIGURE 1:**
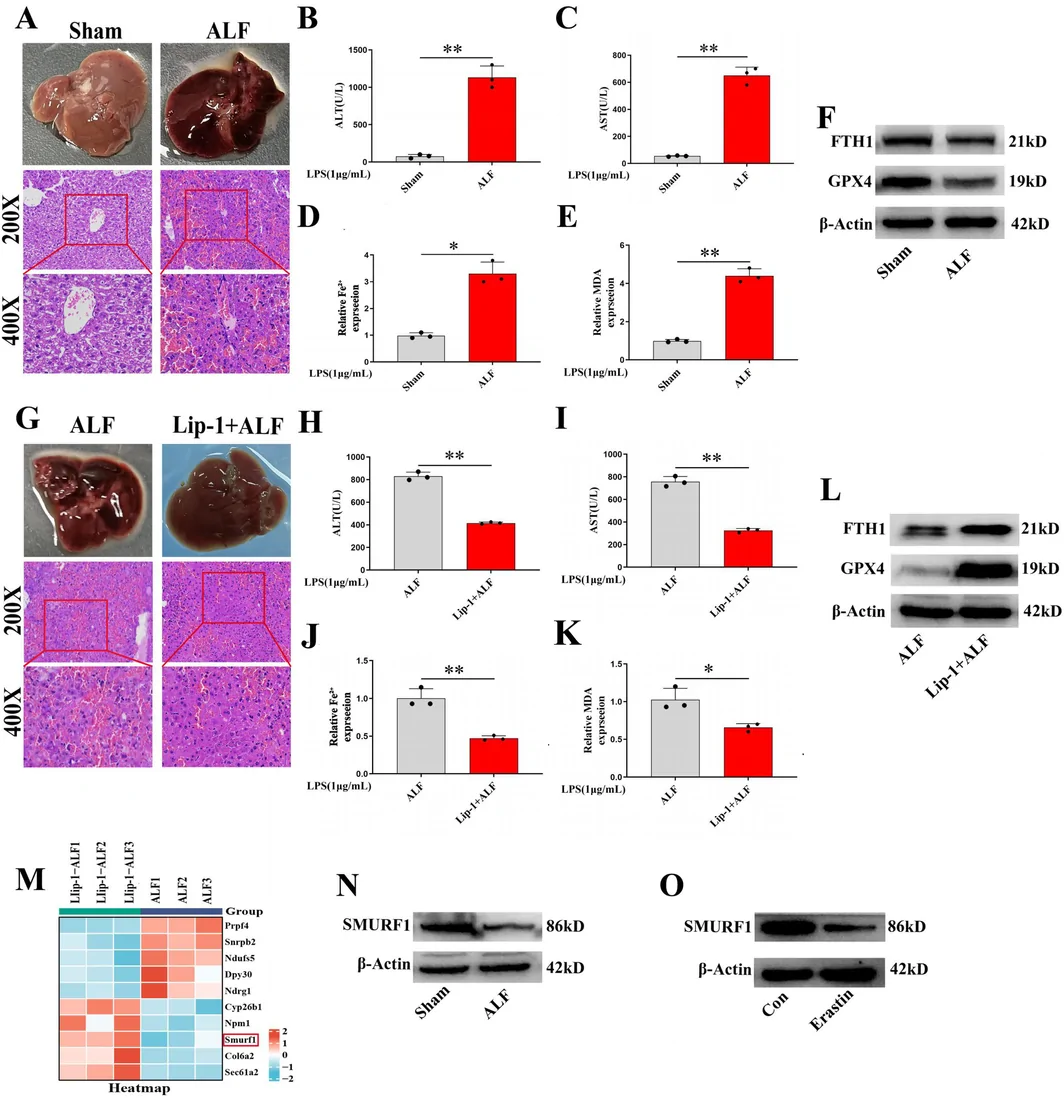
Smurf1 may be a protein associated with ferroptosis in liver injury. (A) Pathological changes in the liver tissue of ALF mice were observed by H&E staining (*n = 3*). (B, C) Measurement of ALT and AST levels in ALF mice (*n* = 3). (D, E) Measurement of Fe^2+^ and MDA concentrations in ALF mice (*n* = 3). (F) The protein expression levels of Fth1 and Gpx4 in ALF mice were detected (*n* = 3). (G) Pathological changes in liver tissues of ALF mice with or without Lip‐1 treatment were observed by H&E staining (*n* = 3). (H, I) Levels of ALT and AST in ALF mice with or without Lip‐1 treatment were measured (*n* = 3). (J, K) Concentrations of Fe^2+^ and MDA in ALF mice with or without Lip‐1 treatment were determined (*n* = 3). (L) Protein expression levels of Fth1 and Gpx4 in ALF mice with or without Lip‐1 treatment were detected (*n* = 3). (M) A volcano plot was used to show the relative protein expression levels in liver tissues between the Lip‐1 group and the ALF group. Proteins marked with red and blue dots represent significantly differentially expressed proteins. (N, O) Protein expression levels of Smurf1 in liver tissues and hepatocyte lines were detected by Western blotting (*n* = 3). *p < 0.05, *p < 0.01.

Subsequently, we intervened in mice with liver failure using the ferroptosis inhibitor Liproxstatin‐1 (Lip‐1). The results showed that Lip‐1 administration significantly improved the progression of liver failure and effectively reduced the level of ferroptosis in liver tissues (Figure 1G–L) This finding further confirms the key role of ferroptosis in the occurrence and development of liver failure, suggesting that elucidating the ferroptosis‐related molecular mechanisms in liver failure is of great significance for the prevention of liver failure.

To this end, we performed proteomic analysis on the liver tissues of Lip‐1‐administered mice and plotted the top five upregulated and downregulated genes into a heatmap (Figure 1M). Recent studies have shown that the ubiquitination modification process plays an important role in liver failure‐related cell death mechanisms, and E3 ubiquitin ligases and deubiquitinating enzymes (DUBs) are also considered important protein targets for improving acute liver failure. Interestingly, we found that the E3 ubiquitin ligase Smurf1 in the heatmap was significantly upregulated after Lip‐1 administration, suggesting that it may play a key role in liver failure‐related ferroptosis.

To further verify the above findings, we detected the expression of Smurf1 in liver tissues and HepaRG cells: in the acute liver failure mouse model, the expression level of Smurf1 was significantly lower than that in the normal control group (Figure 1N); while in the HepaRG cell ferroptosis model, the protein expression of Smurf1 was also significantly reduced (Figure 1O). These data indicate that Smurf1 is downregulated in the state of liver failure and may play an important regulatory role in the process of hepatocyte ferroptosis.

### 3.2 Knockdown of Smurf1 Promotes Hepatocyte Ferroptosis and Aggravates Liver Failure

To determine whether Smurf1 is directly involved in the ferroptosis process, we constructed stable HepaRG cell lines with Smurf1 overexpression or knockdown (Figure 2A; Table 1) and examined the regulatory effect of Smurf1 on hepatocyte ferroptosis. The results showed that after Smurf1 knockdown, in the erastin‐induced hepatocyte ferroptosis model, the Fe^2+^ concentration and MDA content increased, while the expression levels of the anti‐ferroptotic proteins Slc7a11 and Fth1 significantly decreased; conversely, the opposite results were observed in hepatocytes with Smurf1 overexpression (Figure 2B–D). Detection of cell viability via live/dead staining revealed that Smurf1 knockdown promoted erastin‐induced hepatocyte death (Figure 2E,F). Furthermore, a mouse model of acute liver failure with Smurf1 knockdown was successfully established using hepatocyte‐specific AAV‐associated virus (AAV‐sh Smurf1) (Figure 2G). It was further found that Smurf1 knockdown exacerbated liver failure on mouse model (Figure 2H–J). These results suggest that Smurf1 knockdown may exacerbate the progression of liver failure by promoting hepatocyte ferroptosis.

**FIGURE 2:**
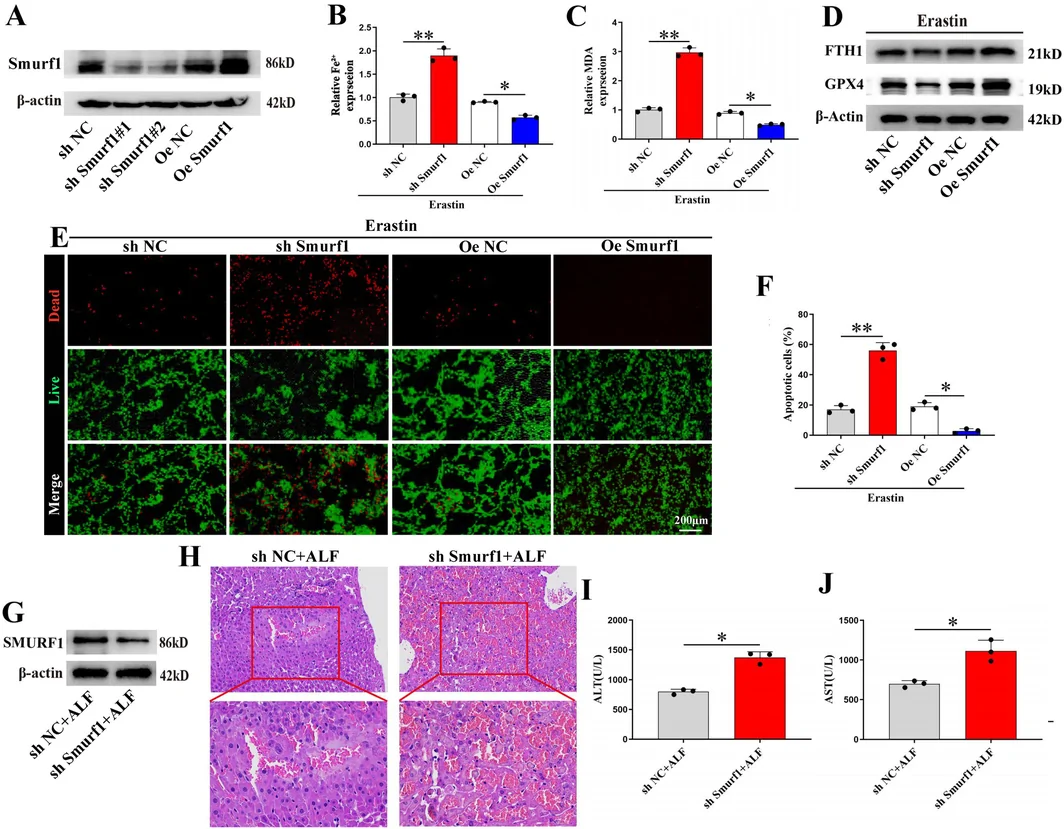
Knockdown of Smurf1 promotes hepatocyte ferroptosis and aggravates liver failure. (A) HepaRG cells were transfected with lentiviruses expressing sh‐Smurf1 or Oe‐Smurf1. Transfection efficiency was detected by western blotting (*n* = 3). (B, C) Hepatocytes with Smurf1 overexpression or knockdown were treated with Erastin (10 μM) for 24 h, and the levels of ferroptosis‐related indicators (Fe^2+^ and MDA) were detected (*n* = 3). (D) Determination of key proteins against ferroptosis (Fth1 and Gpx4) in hepatocytes (*n* = 3). (E, F) Hepatocytes with Smurf1 overexpression or knockdown were treated with Erastin (10 μM) for 24 h; cell viability was detected by live/dead staining (*n* = 3). (G) Western blotting was used to analyse the transfection efficiency of AAV‐sh Smurf1 in ALF mice (*n* = 3). (H) Pathological changes in liver tissue were observed by H&E staining (*n* = 3). (I, J) Determination of ALT and AST in mice (*n* = 3). *p < 0.05, **p < 0.01.

### 3.3 Smurf1 Interacts With Novel Substrate Ulk1 Protein and Promotes the Ubiquitin‐Dependent Degradation of Ulk1

To clarify the specific molecular mechanism by which Smurf1 regulates hepatocyte ferroptosis and identify the downstream target genes of Smurf1, we detected proteins interacting with Smurf1 through IP/MS, predicted substrate proteins that can bind to Smurf1 from ubiquitinase data (http://ubibrowser.bio‐it.cn) and searched for ferroptosis‐related gene from ferroptosis‐associated disease database (http://www.zhounan.org). A cross‐analysis of the three sets of results ultimately identified Ulk1, a ubiquitination modification‐related regulatory protein involved in cellular ferroptosis (Figure 3A). Co‐IP experiments showed that Smurf1 can interact with Ulk1 in hepatocytes (Figure 3B). Further experiments showed that knockdown or overexpression of Smurf1 led to an increase or decrease in Ulk1 protein levels, respectively, with no significant change at the transcriptional level (Figure 3C,D), suggesting that Smurf1 may promote Ulk1 degradation through post‐translational modification. After blocking Ulk1 protein synthesis with cycloheximide (CHX), we found that overexpression of Smurf1 still enhanced Ulk1 degradation (Figure 3E). To clarify the degradation pathway of Ulk1, we treated hepatocytes with the proteasome inhibitor MG132, and the results showed that MG132 could reverse the Smurf1‐mediated decrease in Ulk1 protein levels (Figure 3F), indicating that Smurf1 regulates Ulk1 protein stability through the ubiquitin‐proteasome pathway. Subsequent detection of the effect of Smurf1 on Ulk1 ubiquitination levels revealed that knockdown of Smurf1 significantly reduced Ulk1 ubiquitination levels in hepatocytes and maintained its protein stability, whereas overexpression of Smurf1 significantly increased Ulk1 ubiquitination levels and promoted its protein degradation (Figure 3G). Immunohistochemical (IHC) analysis was conducted to evaluate hepatic Ulk1 expression. Compared with sham mice, ALF mice exhibited markedly increased Ulk1 expression, suggesting that Ulk1 upregulation is associated with ALF pathogenesis (Figure 3H). These results confirm that Smurf1 can directly bind to Ulk1 protein and mediate its ubiquitin‐dependent degradation.

**FIGURE 3:**
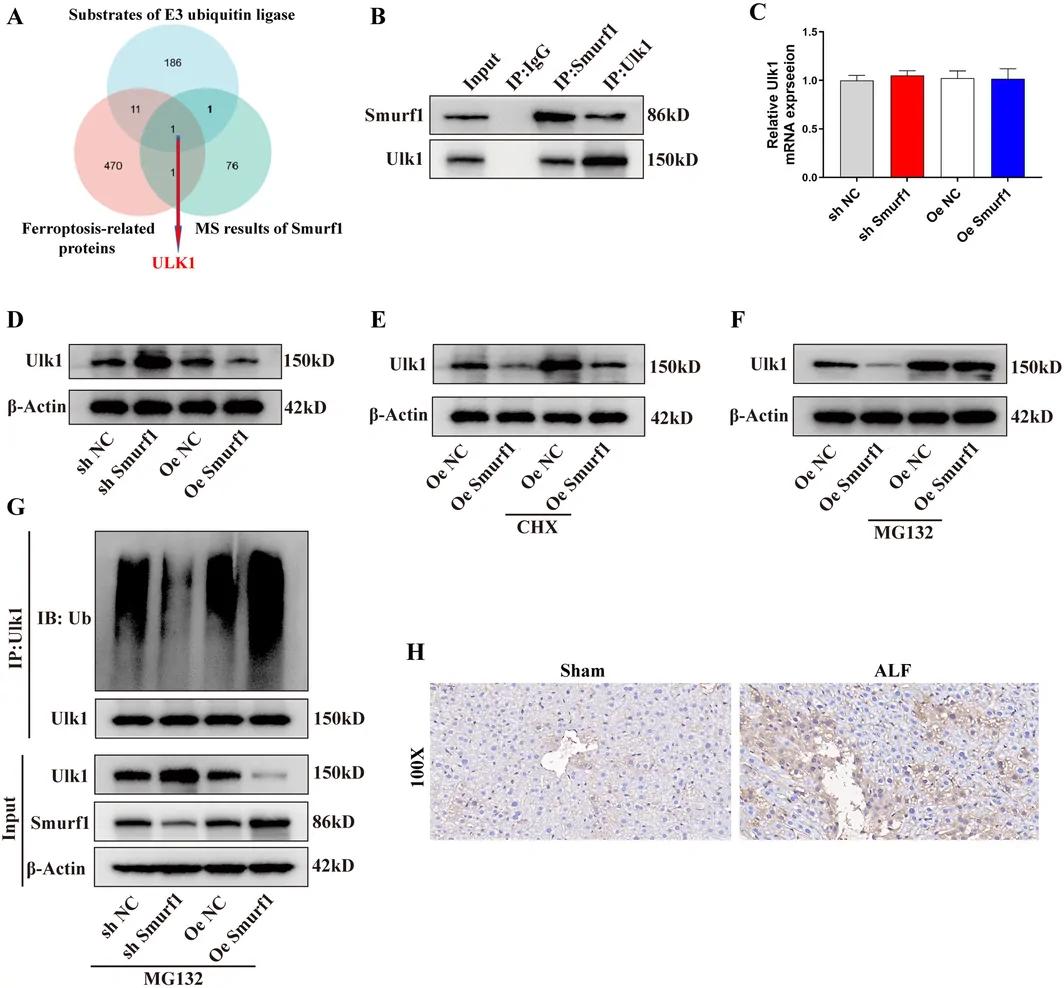
Smurf1 interacts with Ulk1. (A) Interacting proteins between Smurf1's interacting substrates and ferroptosis disease‐associated genes were derived using IP/MS results of Smurf1 and bioinformatics online analysis. (B) Co‐IP experiments was used to detect the interaction between Smurf1 and Ulk1 in hepatocytes (*n* = 3). (C, D) Detect the mRNA and protein levels of Ulk1 in hepatocytes with Smurf1 knockdown and overexpression (*n* = 3). (E) HepaRG cells expressing Oe‐NC or Oe‐Smurf1 were treated with cycloheximide (CHX, 10 μg/mL) for 24 h, followed by detection of Ulk1 protein levels via Western blotting (*n* = 3). (F) In Smurf1‐overexpressing HepaRG cells treated with MG132 (20 μM) for 12 h, the expression level of Ulk1 was detected by Western blotting. (G) Ulk1 ubiquitination and Smurf1 protein levels in Smurf1‐overexpressing HepaRG cells were assessed by Western blotting. (H) Differences in hepatic Ulk1 expression between ALF model mice and normal mice were visualized via IHC staining (*n* = 3).

### 3.4 Knockdown of Smurf1 Promotes Hepatocyte Ferroptosis via Ulk1‐Mediated Autophagy

Studies have shown that Ulk1 is recognized as an initiator of autophagy, which can induce autophagy activation; conversely, inactivation of Ulk1 also leads to the termination of autophagy. Given that Smurf1 can regulate the stability of Ulk1, we evaluated the effect of Smurf1 on autophagic flux in hepatocytes. The results demonstrated that Smurf1 inhibited the expression of LC3II and increase the protein level of P62, whereas Smurf1 knockdown exerted the opposite effect (Figure 4A). These findings suggested that Smurf1 knockdown activated the autophagic level in hepatocytes. It has been reported that excessive activation of cellular autophagy leads to iron accumulation by degrading ferritin, thereby promoting cellular ferroptosis. Therefore, ferroptosis is regarded as a downstream process of cellular autophagy. We further assessed the role of the cellular autophagic process in Smurf1‐regulated hepatocyte ferroptosis. The results indicated that Smurf1 knockdown activated autophagy and ferroptosis levels in hepatocytes, while Ulk1 knockdown reversed the promoting effect of Smurf1 knockdown on hepatocyte ferroptosis (Figure 4B–E; Table 1). The above results suggest that Smurf1 knockdown promotes hepatocyte ferroptosis depending on Ulk1‐mediated autophagy.

**FIGURE 4:**
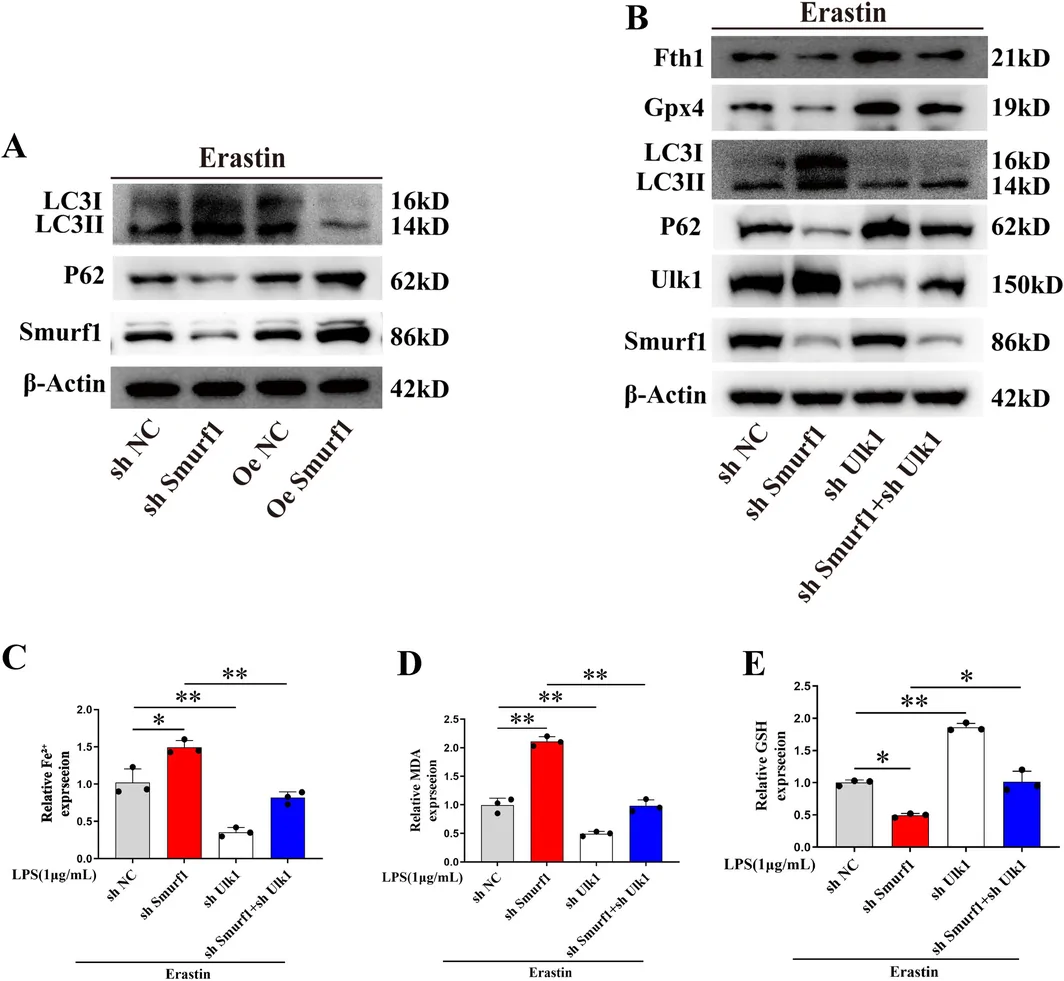
Knockdown of Smurf1 promotes hepatocyte ferroptosis via Ulk1‐mediated autophagy. (A) HepaRG cells were transfected with Oe‐Smurf1 and sh‐Smurf1 lentiviruses, respectively and then treated with Erastin (10 μM) for 24 h to detect the protein expression levels of autophagy‐related proteins (LC3I, LC3II and P62) (*n* = 3). (B) HepaRG cells were transfected with Sh‐Smurf1 and Sh‐Ulk1 + Sh‐Smurf1 lentiviruses, respectively and then treated with Erastin (10 μM) for 24 h to detect the protein expression levels of autophagy‐related proteins (LC3I, LC3II and P62) and ferroptosis‐related proteins (Fth1 and Gpx4) (*n* = 3). (C–E) HepaRG cells were transfected with Sh‐Smurf1 and Sh‐Ulk1 + Sh‐Smurf1 lentiviruses, respectively, and then treated with Erastin (10 μM) for 24 h to detect ferroptosis levels (*n* = 3). *p < 0.05, **p < 0.01.

## 4 Discussion

Preliminary studies by our research group have shown that ferroptosis occurs in hepatocytes of mice with acute liver failure, and ferroptosis may be an important mechanism for regulating the progression and alleviation of liver failure. In this study, we found that E3 ubiquitin ligase Smurf1, an autophagy‐dependent ferroptosis‐related protein, when knocked down, promotes ferroptosis in hepatocytes and exacerbates the progression of liver failure. Mechanistically, Smurf1 directly targets and binds to Ulk1, thereby promoting the ubiquitin‐dependent degradation of Ulk1. After Smurf1 knockdown, the ubiquitination level of Ulk1 is reduced, which maintains Ulk1 protein homeostasis. This leads to increased Ulk1‐mediated autophagy, promotes hepatocyte ferroptosis, and consequently exacerbates acute liver failure. Therefore, the discovery of the Smurf1‐mediated Ulk1‐autophagy pathway provides new insights into ferroptosis in liver failure.

Smurf1 regulates a variety of important biological pathways, such as bone homeostasis, cell migration and pathogenic autophagy, by recognizing and binding to substrate proteins and influencing their ubiquitination [17, 18, 30, 31]. A growing number of studies have reported that Smurf1 can affect cell survival by regulating the ubiquitination of proteins associated with ferroptosis. For instance, Xiong et al. found that Smurf1 maintains skeletal muscle health by promoting FTH1 ubiquitination and subsequent degradation to regulate iron homeostasis [32]. Smurf1 may promote neuronal necrosis and ferroptosis after LPS‐induced neuroinflammation [33]. In addition, Smurf1 activates NRF2 by promoting the ubiquitination and degradation of GDF15, thereby inhibiting ferroptosis in Alzheimer's disease [34]. In this study, we confirmed the presence of ferroptosis in acute liver failure. Further proteomic analysis and experimental verification showed that Smurf1 was downregulated in acute liver failure and was mainly expressed in hepatocytes. These results suggest that Smurf1 may be a ferroptosis‐related protein in liver failure. However, ferroptosis of hepatocytes after liver failure is a complex process, and its molecular mechanism requires further in‐depth study.

Iron ion‐catalysed polyunsaturated fatty acids (PUFAs) lead to an increase in lipid peroxides, and the lipotoxic environment causes the accumulation of reactive oxygen species (ROS) and oxidative stress, thereby triggering ferroptosis [4]. Inhibitors of ferroptosis and their upstream metabolic regulation can serve as powerful therapeutic targets for ALF [35, 36, 37, 38]. Consistent with previous research findings, we observed that Smurf1 inhibited ferroptosis in hepatocytes and alleviated the progression of liver failure. Subsequently, we explored the mechanism by which Smurf1 knockdown exacerbates ferroptosis in hepatocytes and identified Ulk1 as a substrate and interacting protein of Smurf1 in hepatocytes. In ubiquitination modification, ubiquitin chains with different linkage types perform distinctly different biological functions. The K48‐linked and K63‐linked ubiquitin chains are classic signals that mediate the degradation and stabilization of target proteins, respectively [39, 40, 41]. Given that Smurf1 can ubiquitinate Ulk1, we conducted ubiquitination experiments to clarify whether this modification ultimately leads to the degradation or increased stability of Ulk1 protein. The results showed that Smurf1 can significantly promote the degradation of Ulk1 protein by catalysing the formation of K48‐linked ubiquitin chains on Ulk1.

Ulk1 is the promoter of autophagy and has the biological function of positively regulating autophagy. It is related to different pathophysiological processes and is of great significance for maintaining the homeostasis of eukaryotic cells [42]. Ulk1 is a key regulator of apoptosis and autophagy and may affect ferroptosis, although its exact mechanism has not yet been fully defined [43, 44, 45]. Studies have shown that Ulk1 promotes cardiac hypertrophy through ferroptosis in cardiomyocytes mediated by the HMGA2/METTL14/SLC7A11 axis [46]. Acrylamide promotes cell death by activating the AMPK‐ULK1‐MTOR signalling in human chondrocytes to induce autophagy‐dependent ferroptosis [47]. Autophagy is considered an upstream mechanism of ferroptosis, and it remains unclear whether the cellular autophagic process plays a role in the regulation of hepatocyte ferroptosis by Smurf1. Here, we found that knockdown or overexpression of Smurf1 activated or inhibited the autophagic flux level of hepatocytes, respectively. To further explore whether Ulk1‐mediated autophagy is involved in the regulation of hepatocyte ferroptosis by Smurf1, we observed that knockdown of Smurf1 significantly promoted the activation of Ulk1‐mediated autophagy and the level of ferroptosis in hepatocytes, while the knockdown of Ulk1 reversed the pro‐ferroptotic effect exerted by knockdown of Smurf1. These results suggest that Smurf1 knockdown exerts a role in promoting hepatocyte ferroptosis dependent on the activation of Ulk1‐mediated autophagy.

In conclusion, we identified a ferroptosis‐related protein, Smurf1, which regulates hepatocyte ferroptosis in liver failure by targeting Ulk1 to mediate autophagy. Mechanistically, we demonstrated that the E3 ligase Smurf1 interacts with its novel substrate Ulk1 and enhances K63‐linked polyubiquitin chains, thereby increasing the ubiquitination level and stability of Ulk1, which in turn leads to elevated autophagy initiated by Ulk1. Since autophagy is the upstream of ferroptosis, our findings highlight that Smurf1 knockdown inhibits the ubiquitin‐dependent degradation of Ulk1, thereby activating autophagy to degrade ferritin and release a large amount of free iron, leading to abnormal accumulation of ROS and inducing ferroptosis of hepatocytes, which aggravates the progression of acute liver failure. The Smurf1‐Ulk1 axis may serve as a potential therapeutic target for regulating ferroptosis in liver failure. A schematic description of Smurf1‐Ulk1 axis on acute liver failure is expounded in Figure 5.

**FIGURE 5:**
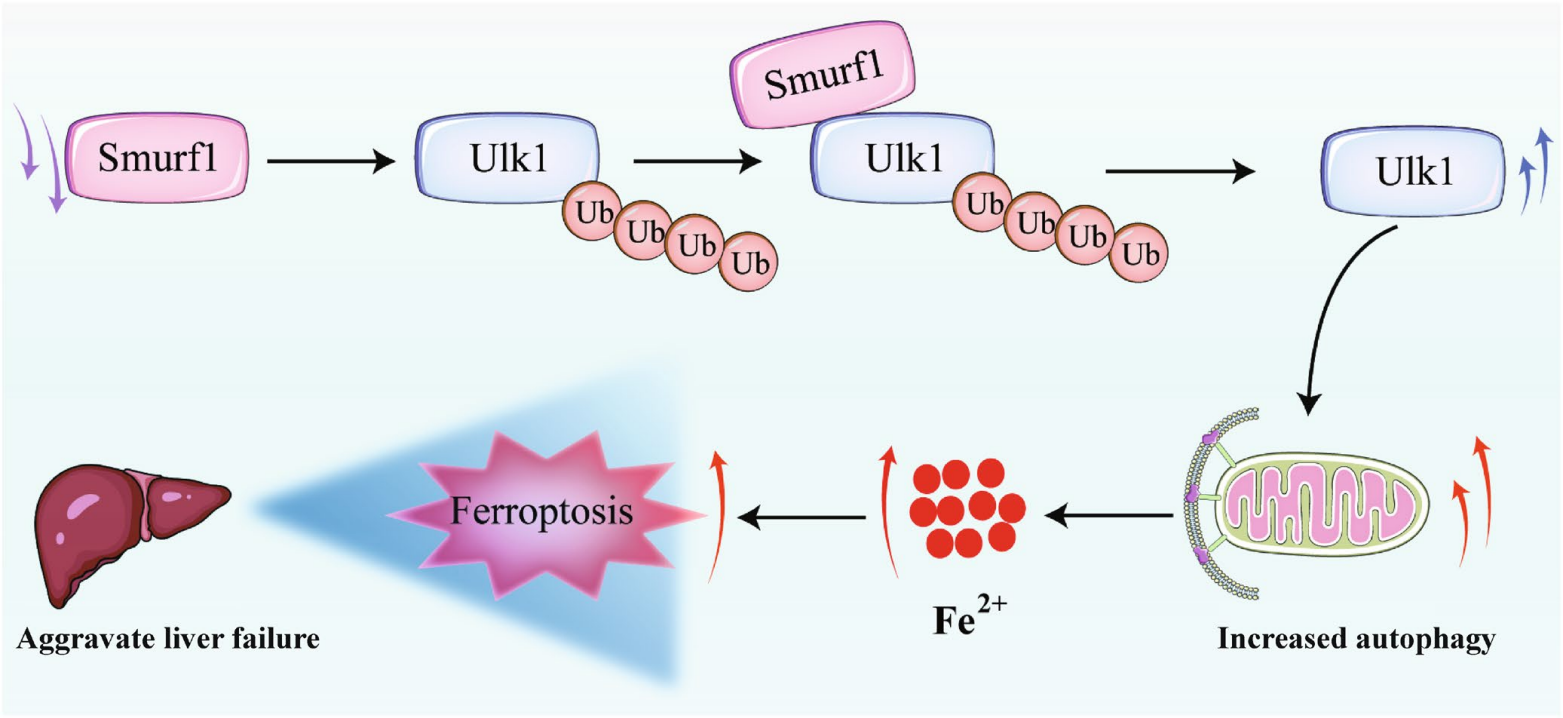
A schematic description of the Smurf1‐Ulk1 axis on acute liver failure. Knockdown of Smurf1 promotes autophagy‐dependent ferroptosis in acute liver failure by inhibiting ubiquitin‐mediated Ulk1 degradation.

## Author Contributions

**Pei Shi:** investigation, methodology, writing – original draft. **Mengyu Tao:** conceptualization, formal analysis. **Yuyu Zeng:** methodology, data curation. **Yuchen Peng:** formal analysis, data curation. **Xiaoping Wu:** project administration, writing – review and editing.

## Funding

This work was supported by National Natural Science Foundation of China, 82560133.

## Conflicts of Interest

The authors declare no conflicts of interest.

## Data Availability

The data that support the findings of this study are available from the corresponding author upon reasonable request.
